# Perioperative versus adjuvant chemotherapy for resectable gastric cancer: a meta-analysis of randomized controlled trials

**DOI:** 10.3389/fonc.2025.1432596

**Published:** 2025-03-06

**Authors:** Haiya Ou, Jiamei Zhuang, Mingwei Jian, Xinyi Zheng, Tingping Wu, Honghui Cheng, Rui Qian

**Affiliations:** ^1^ Department of Gastroenterology, Shenzhen Bao’an Chinese Medicine Hospital, Guangzhou University of Chinese Medicine, Shenzhen, China; ^2^ Department of Nephrology, The Fourth Clinical Medical College of Guangzhou University of Chinese Medicine, Shenzhen, China

**Keywords:** neoadjuvant chemotherapy, adjuvant chemotherapy, gastric cancer, meta-analysis, NAc

## Abstract

**Objectives:**

To report the latest systematic review and meta-analysis of randomized controlled trials (RCT) to compare perioperative versus adjuvant chemotherapy for resectable gastric cancer.

**Methods:**

We conducted a systematic literature retrieval via PubMed, Embase, Web of Science, and Cochrane until April, 2024 for RCT which compared perioperative versus adjuvant chemotherapy for resectable gastric cancer. Outcomes measured were overall survival (OS) and progression-free survival (PFS).

**Results:**

5 RCTs including 2,735 patients were included for meta-analysis. Meta-analysis revealed a significant longer PFS in the neoadjuvant chemotherapy (NAC) group (HR: 0.77; 95% CI: 0.69, 0.85; *P*<0.00001) compared with adjuvant chemotherapy (AC) group. Subgroup analysis found that there was still a significant superiority of NAC in female (HR: 0.53; 95% CI: 0.40, 0.70; *P*<0.0001) and cN+ (HR: 0.77; 95% CI: 0.67, 0.89; *P*=0.0005) patients, while the superiority disappeared in male (HR: 0.87; 95% CI: 0.74, 1.01; *P*=0.07) and cN- patients (HR: 0.91; 95% CI: 0.46, 1.78; *P*=0.77). In addition, meta-analysis observed a trend towards improved OS with NAC (HR: 0.86; 95% CI: 0.70, 1.07; *P* = 0.17), and sensitivity analysis demonstrated instability in OS.

**Conclusions:**

NAC can significantly prolong PFS in patients with resectable gastric cancer compared to AC, and the benefit is more significant in women and cN+ patients. Besides, our analysis indicated that NAC has a potential to improve OS compared with AC.

**Systematic review registration:**

https://www.crd.york.ac.uk/PROSPERO/, identifier CRD42024546165.

## Introduction

Gastric cancer is the fifth most common malignancy worldwide and the third cause of cancer death (1). China is a country with a high incidence of gastric cancer. In 2015, the incidence and mortality rate of gastric cancer in China ranked second among all malignant tumors, second only to lung cancer (2). At present, the treatment of gastric cancer mainly includes surgery, chemotherapy, radiotherapy, targeted drug therapy and immunotherapy. For locally advanced gastric cancer, D2 radical gastrectomy is the standard operation (3–6), and D2 operation plus adjuvant chemotherapy (AC) is the standard treatment in Asian country, including China, Korea and Japan (7). The ACTS-GC study conducted in Japan in 2007 (8) fully demonstrated the superiority of postoperative AC in prolongating the survival of patients. In this study, 1059 patients with locally advanced gastric cancer who received D2 radical resection were enrolled, and these patients were divided into S-1 single-agent AC group and operation group. The results of this study showed that the 3-year survival rate of the two groups was different, with the 3-year survival rate of the AC group being 72.2% and that of the surgery group being 59.6%. The 3-year survival rate was 12.6% higher in the AC group (HR: 0.62, 95%CI: 0.50-0.77).

At the end of the 20th century, the term neoadjuvant chemotherapy (NAC) was first proposed by Frie (9). NAC refers to chemotherapy after a patient is diagnosed with cancer, before surgery or radiation therapy, also known as preoperative chemotherapy. In this context, perioperative chemotherapy refers to NAC combined with adjuvant chemotherapy. MAGIC study (10) applied perioperative ECF protocol to enrolled patients with resectable gastric cancer and esophagogastric junctional adenocarcinoma, and the results showed that perioperative chemotherapy group significantly improved the long-term survival rate of patients, and the R0 resection rate increased to 79.3%. PRODIGY study (11) included a total of 484 patients with gastric or gastroesophageal junction adenocarcinoma, who were randomly divided into NAC (docetaxel+oxaliplatin+S-1) combined with radical gastrectomy combined with S-1 single-drug AC group (intervention group) and postoperative S-1 single-drug AC group (control group). The results showed that: The 3-year PFS rates in the two groups were 66.3% and 60.2%, respectively (HR: 0.70, 95%CI: 0.52-0.95), which indicated that addition of NAC DOS regimen on the basis of D2 gastrectomy and adjuvant S-1 therapy could improve progression-free survival in patients with advanced gastric cancer. The RESOLVE study (12) published at the same time showed that neoadjuvant SOX chemotherapy could improve the disease-free survival of patients compared with postoperative XELOX chemotherapy. In the other group, the disease-free survival of postoperative SOX chemotherapy regimen was no worse than postoperative XELOX chemotherapy regimen.

The meta-analysis published by Wei et al. (13) included 18 studies, including RCTs and non-randomized clinical trials. The results showed that gastric cancer patients treated with NAC had a longer OS (HR: 0.77, 95%CI: 0.69-0.87) and PFS (HR: 0.76, 95% CI: 0.69-0.84) compare with those receiving AC. Following this, Wang et al. (14) published an RCT with a larger sample size (756 patients), the results of which may change the status quo of NAC and AC treatment for gastric cancer. Therefore, the aim of this paper was to conduct a systematic review and meta-analysis of all existing RCTs to assess the difference in survival benefit between perioperative chemotherapy and AC for patients with resectable gastric cancer.

## Methods

### Literature search

This meta-analysis was performed according to the PRISMA (Preferred Reporting Items for Systematic Reviews and Meta Analysis) 2020 statement (15) and has been registered in the PROSPERO (CRD42024546165). We conducted a systematic literature search via PubMed, Embase, Web of Science, and Cochrane up to April, 2024 for RCT that compared perioperative versus adjuvant chemotherapy for resectable gastric cancer. We searched the literature through the following terms: “neoadjuvant”, “perioperative”, “preoperative”, “gastric cancer”, and “chemotherapy”. The detailed search strategies are as follows: (((Neoadjuvant OR Perioperative OR Preoperative) AND ((“Drug Therapy”[Mesh]) OR (((((Drug Therapies) OR (Chemotherapy)) OR (Chemotherapies)) OR (Pharmacotherapy)) OR (Pharmacotherapies)))) AND ((“Stomach Neoplasms”[Mesh]) OR (((((((((Stomach Neoplasm) OR (Gastric Neoplasms)) OR (Gastric Neoplasm)) OR (Cancer of Stomach)) OR (Stomach Cancers)) OR (Gastric Cancer)) OR (Gastric Cancers)) OR (Stomach Cancer)) OR (Cancer of the Stomach)))) AND (random*). Furthermore, we manually screened the bibliography lists of all included RCTs. Two authors (HYO and JMZ) retrieved and assessed eligible articles independently. Any differences in literature retrieval were resolved by discussion with the third author (RQ).

### Inclusion and exclusion criteria

Articles were eligible when meeting the following standards:

P: patients diagnosed with resectable gastric cancer.

I: perioperative or NAC combined with surgery.

C: postoperative AC combined with surgery.

O: survival outcome, such as overall survival (OS), progression-free survival (PFS), and relapse-free survival (RFS), etc.

S: randomized controlled trials.

We excluded study protocols, unpublished studies, non-original studies (including meeting abstracts, correction, and reply), non-RCT studies, studies without sufficient data (survival data cannot be obtained directly or through data transformation), and reviews.

### Data abstraction

Two authors independently conducted data abstraction, with any differences resolved by a third author. The following information was abstracted from eligible RCTs: first author name, publication year, research period, study region, study design, registration number, population, intervention, control, sample size, age, gender, follow-up time, OS, PFS and subgroup outcomes. If research data were insufficient, corresponding authors were contacted for complete data when available.

### Quality evaluation

The evaluation of the quality of eligible RCT was performed according to the Cochrane Handbook for Systematic Reviews of Interventions 5.1.0, considering seven domains: sequence generation randomization, allocation concealment, blinding of participants and personnel, outcome assessment blinding, incomplete outcome data, selective outcome reporting, and other potential sources of bias (16). Each study aspect was assigned one of three evaluation outcomes: low risk, high risk, or unclear risk. Studies with more “low risk” bias evaluations were considered superior. Two authors independently assessed the quality of all included studies, resolving any disagreements through discussion.

### Statistical analysis

The synthesis of data was performed utilizing Review Manager 5.4.1. For the evaluation of survival outcomes, hazard ratios accompanied by 95% confidence intervals were employed. The assessment of heterogeneity across outcomes was conducted through the application of the chi-squared (χ^2^) test (Cochran’s Q) and the inconsistency index (17). Substantial heterogeneity was characterized by a χ^2^ P value below 0.1 or an *I* ^2^ exceeding 50%. The computation of the overall HR was performed utilizing the random-effects model. When data were adequate, subgroup analyses based on gender, cT stage, and cN stage were conducted for survival outcomes to assess potential confounding factors. For results encompassing more than two included studies, a sensitivity analysis was carried out to evaluate the impact of each individual RCT on the overall HR. The assessment of publication bias was performed through Egger’s regression tests (18) through Stata 15.1 edition (Stata Corp, College Station, Texas, USA). *P* value < 0.05 was considered as statistically significant publication bias.

## Results

### Literature retrieval, study characteristics, and baseline

**Figure 1:**
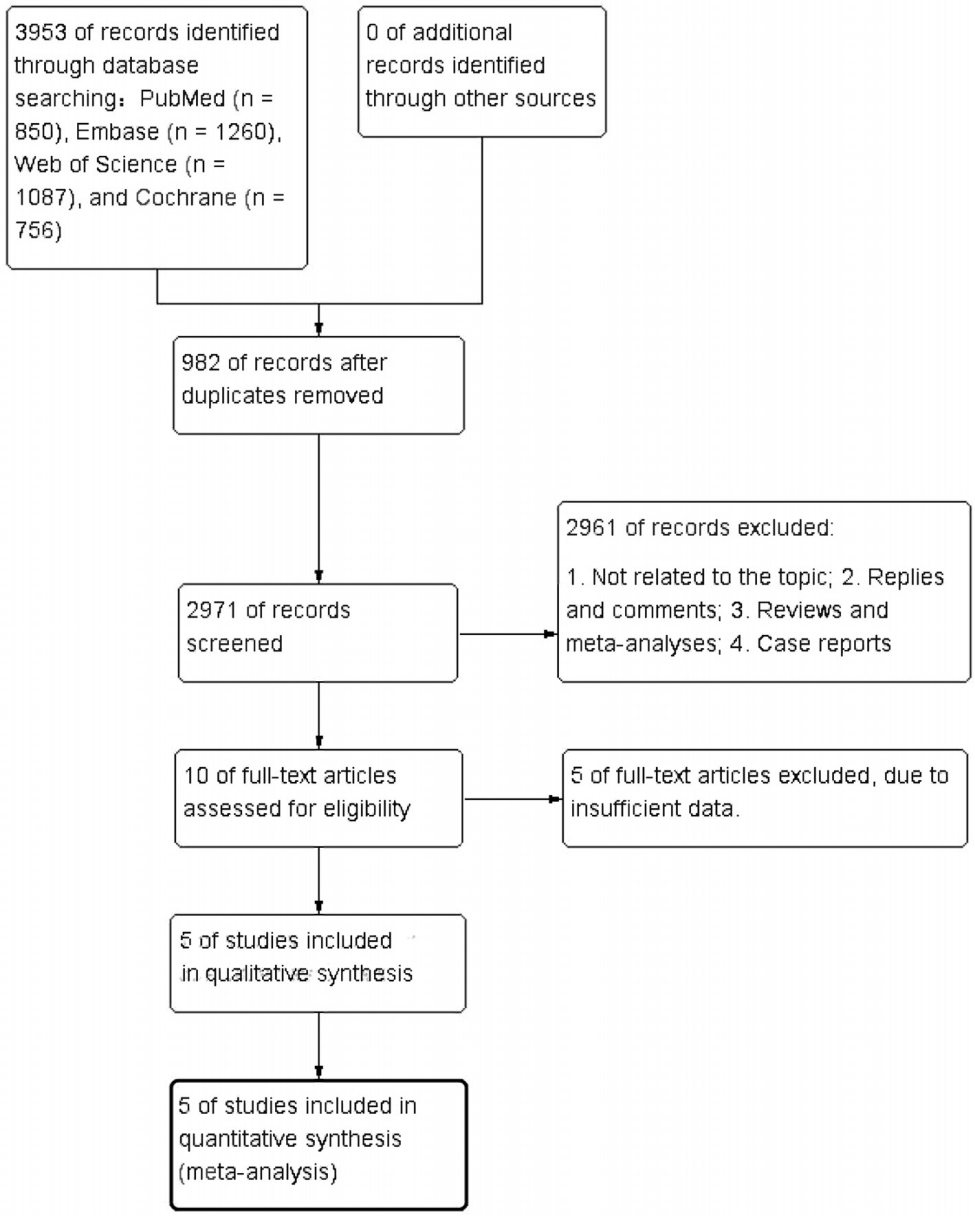
Flowchart of the systematic search and selection process.

**Figure 2:**
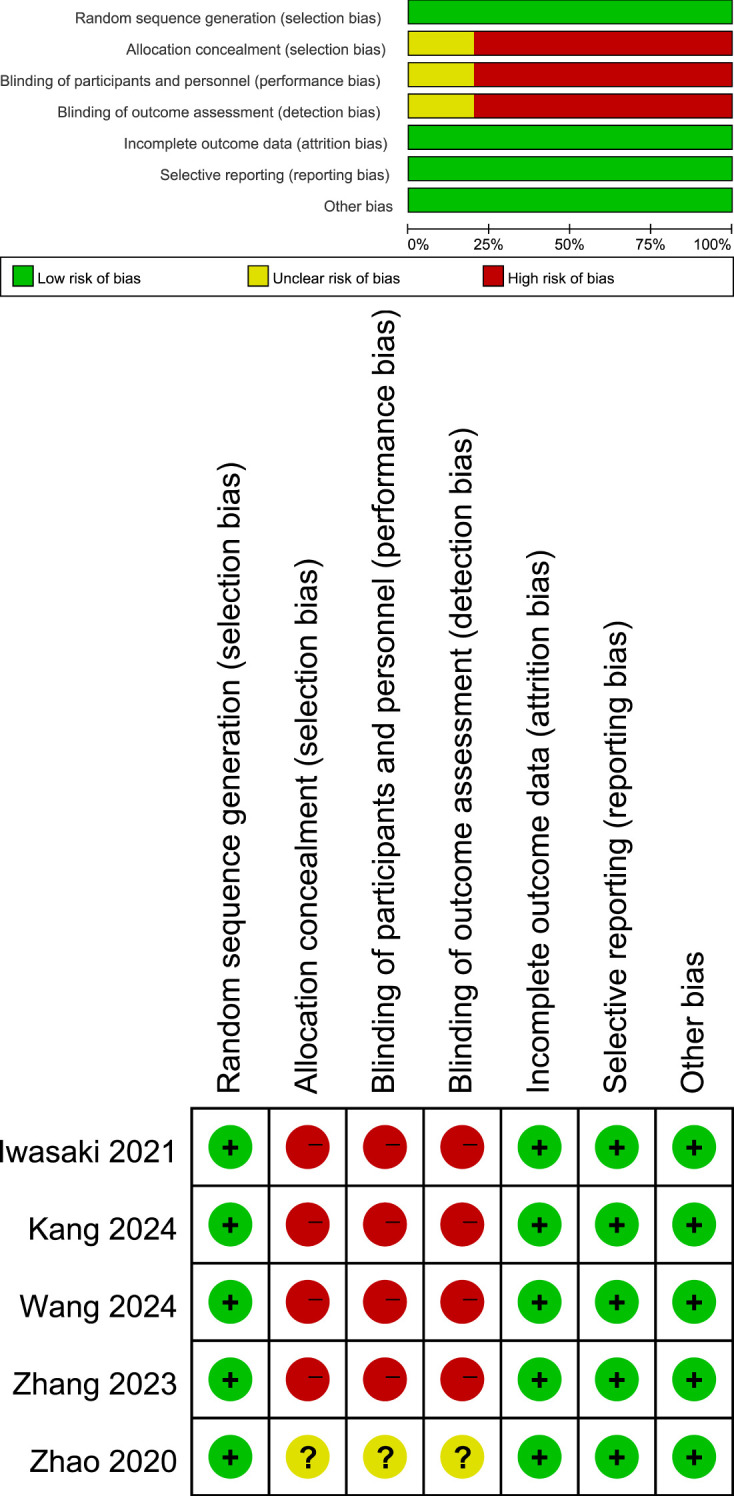
Details of the quality evaluation for included RCTs. Green (+) represents high risk, yellow ()? represents unclear risk, and red (-) represents high risk.

**Figure 1** shows the flowchart of the literature retrieval and selection process. A total of 3,953 related studies in PubMed (n = 850), Embase (n = 1,260), Web of Science (n = 1,087), and Cochrane (n = 756) were identified via systematically literature search. After removing duplicate studies, a total of 2,971 titles and abstracts were evaluated. Of these, two studies were excluded during rescreening due to non-randomized controlled study design and the wrong population (19, 20). Eventually, 5 RCTs including 2,735 patients were included for meta-analysis. **Table 1** presents the characteristics of each eligible RCT. Details of the quality evaluation for all included RCTs are shown in **Figure 2**.

**Table 1:** Baseline characteristics of include studies.

Authors	Study period	Country	Study design	Registration number	Population	Intervention	Control	Median follow-up	Definition of PFS	Patients (n)	Mean/median age	Gender (male)
NAC/AC	NAC/AC	NAC/AC
Iwasaki 2021 (21)	2005-2013	Japan	Open-label, phase 3, randomized controlled trial	UMINCTR (No. C000000279)	Patients aged 20–75 years without a macroscopic unresectable factor as confirmed via staging laparoscopy	NAC (S-1plus cisplatin) followed by D2 gastrectomy plus adjuvant chemotherapy with S-1	Surgery followed by adjuvant chemotherapy with S-1	4.5 years	The time from randomization to the first occurrence of disease progression confirmed by clinical or image diagnosis, such as progression before surgery, diagnosis of being unable to undergo R0 or R1 resection even when a progression-free status was verified, or death from any cause	151/149	64/62	87/89
Kang 2024 (22)	2012-2022	Korea	Phase III RCT	NCT01515748	Patients 20-75 years of age, with EasternCooperativeOncology Group performance status 0-1, and with histologically confirmed primary gastric or gastroesophageal junction adenocarcinoma (clinical TNM staging: T2-3N1 or T4Nany)	Neoadjuvant DOS (docetaxel 50 mg/m2 100 mg/m2, oxaliplatin intravenously day 1, S-1 40 mg/m2 orally twice a day, days 1-14 every 3 weeks for three cycles) before D2 surgery, followed by adjuvant S-1 (CSC group)	D2 surgery followed by adjuvant S-1 (40-60mg orally twice a day, days 1-28 every 6 weeks for eight cycles; SC group)	99.5 months	PD or death, with PD defined as follows (1): in the CSC arm only, RECIST PD during neoadjuvant chemotherapy, and (2) in both the CSC and SC arms, (a) finding of distant metastasis or reporting of distant metastasis from pathology irrespective of intraoperative curative resection; (b) persistence of visually observed cancer cells at resection margin (R2) or microscopic cancer cells at resection margin from postoperative histology (R1) that could not be further removed; or (c) recurrence, either local or at distant sites, during follow-up after R0 resection	238/246	58/58	184/200
Wang 2024 (14)	2012-2019	China	Randomized, open-label, phase 3 trial	NCT01583361	Stage II/III resectable gastric cancer	Two to four cycles of SOX followed by surgery and four to six cycles of SOX	Upfront surgery and eight cycles of SOX	NA	The period from randomization to any recurrence, new cancer, metastases, death, or evident progression for patients who did not receive surgery	382/374	60/59	276/279
Zhang 2023 (23)	2012-2017	China	Open-label, superiority and non-inferiority, phase 3 randomised controlled trial	NCT01534546	Antitumour treatment-naive patients aged 18 years or older with historically confirmed cT4a N+ M0 or cT4b Nany M0 gastric or gastro-oesophageal junction adenocarcinoma	Perioperative SOX (intravenous oxaliplatin 130 mg/m² on day one of each 21 day plus oral S-1 40–60 mg twice a day for three cycles preoperatively and five cycles postoperatively followed by three cycles of S-1 monotherapy)	Adjuvant CapOx (eight postoperative cycles of intravenous oxaliplatin 130 mg/m² on day one of each 21 day cycle plus oral capecitabine 1000 mg/m² twice a day)	62.8 months	The time from randomization to the recurrence of primary cancer, new gastric cancer, distant metastases (assessed by each investigator), or death from any cause, whichever came first. For patients who did not undergo radical gastrectomy, PFS was defined as the time from randomization to overt disease progression	337/345	60/59	271/259
Zhao 2020 (24)	2011-2016	China	Phase III randomized, multicenter, trial	NCT01516944	Patients with advanced gastric cancer	Perioperative S-1 plus oxaliplatin	Adjuvant S-1 plus oxaliplatin	20.6 months	The time began from the gastrectomy to disease recurrence or death by any causes	223/290	NA	177/219

PFS, progression-free survival; NAC, neoadjuvant chemotherapy; AC, adjuvant chemotherapy; RCT, randomized controlled trials.

### PFS

**Figure 3:**
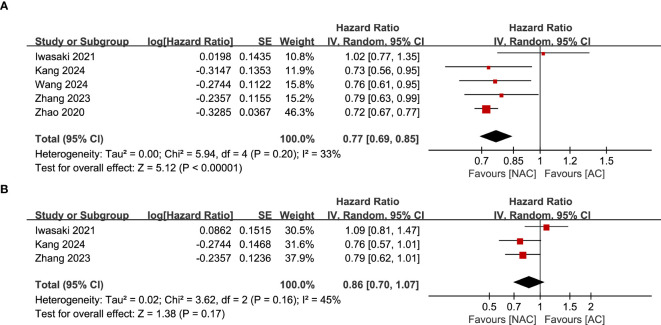
Forest plots of **(A)** PFS, **(B)** OS.

**Figure 4:**
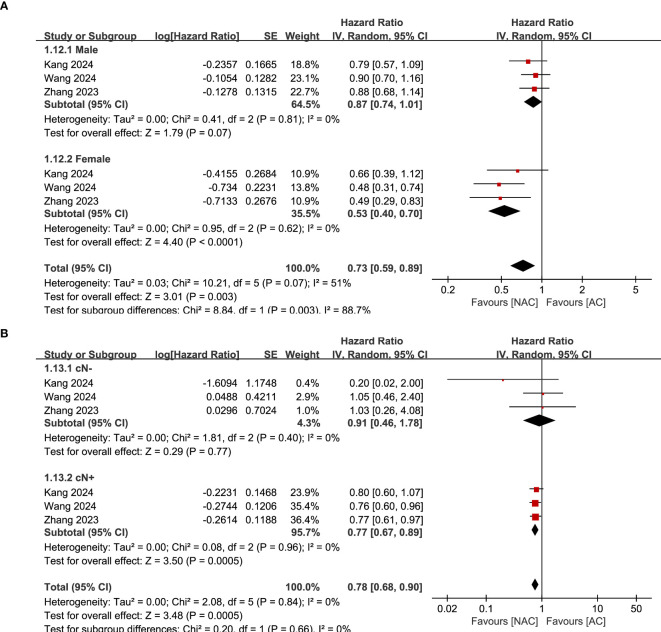
Subgroup analysis of PFS based on **(A)** gender and **(B)** cN stage.

Results of PFS were synthesized from 5 RCTs including 2,735 patients (12, 14, 21–24). Meta-analysis revealed a significant longer PFS in the NAC group (HR: 0.77; 95% CI: 0.69, 0.85; *P*<0.00001) without significant heterogeneity (*I* ^2^ = 33%, *P*=0.20) (**Figure 3A**). Subgroup analysis found that there was still a significant superiority of NAC in female (HR: 0.53; 95% CI: 0.40, 0.70; *P*<0.0001) and cN+ (HR: 0.77; 95% CI: 0.67, 0.89; *P*=0.0005) patients, while the superiority disappeared in male (HR: 0.87; 95% CI: 0.74, 1.01; *P*=0.07) and cN- patients (HR: 0.91; 95% CI: 0.46, 1.78; *P*=0.77) (**Figure 4**) (**Table 2**).

**Table 2:** Subgroup analysis of PFS and OS.

Subgroup	PFS				OS			
	Study	HR [95%CI]	*P* value	*I* ^2^	Study	HR [95%CI]	*P* value	*I* ^2^
**Total**	5	0.77 [0.69-0.85]	<0.00001	30%	3	0.86 [0.70-1.07]	0.17	45%
Gender								
Male	3	0.87 [0.74-1.01]	0.07	0%	2	0.90 [0.63-1.28]	0.56	47%
Female	3	0.53 [0.40-0.70]	<0.0001	0%	2	0.94 [0.67-1.31]	0.71	0%
cT stage								
T1-T3	/	/	/	/	2	1.16 [0.89-1.51]	0.28	0%
T4	/	/	/	/	1	0.69 [0.51-0.95]	0.02	/
cN stage								
N-	3	0.91 [0.46-1.78]	0.77	0%	1	0.38 [0.04-3.44]	0.40	/
N+	3	0.77 [0.67-0.89]	0.0005	0%	2	0.93 [0.67-1.29]	0.68	53%

### OS

Data synthesis OS was performed in 3 RCTs including 1,466 patients (11, 21, 23). Meta-analysis observed a similar OS between the NAC and AC group (HR: 0.86; 95% CI: 0.70, 1.07; *P* = 0.17) without significant heterogeneity (*I* ^2^ = 45%, *P* = 0.16) (**Figure 3B**). Subgroup analysis found that the difference of OS in the two groups remained non-significant in male, female, cN+, cN- and cT1-T3 stage patients, but changed to significant in the cT4 stage patients (**Table 2**).

### Sensitivity analysis and publication bias

**Figure 5:**
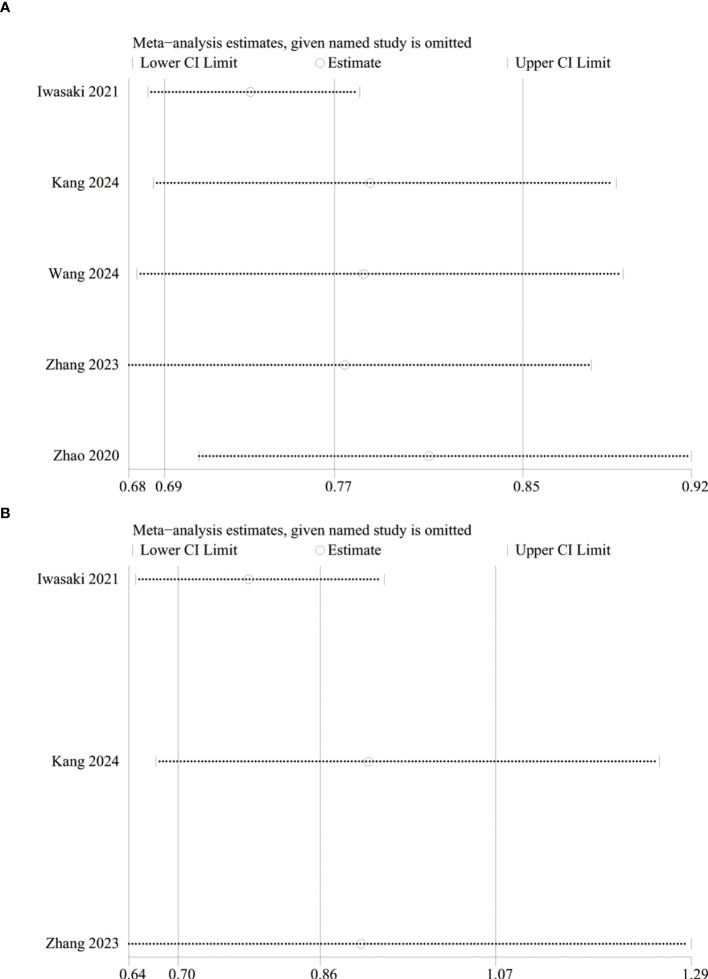
Sensitivity analysis of PFS **(A)** and OS **(B)**.

We performed sensitivity analysis for the results of PFS and OS to assess the effect of each RCT on the total HR via excluding eligible RCTs one by one. Sensitivity analysis found that the new total HR kept stable after removing of each RCT for PFS (**Figure 5A**). However, when Iwasaki’s (21) data were excluded, the difference of OS changed from nonsignificant to significant (HR: 0.78; 95% CI: 0.65, 0.94; *P* = 0.008), and the heterogeneity decreased to 0%, suggesting that perioperative chemotherapy can significantly prolong the OS of patients compared with adjuvant chemotherapy (**Figure 5B**). In addition, the Egger’s test of PFS (*P*=0.184) and OS (*P*=0.620) did not detect a potential publication bias.

## Discussion

Gastric cancer is one of the most common malignant tumors in clinical practice, which seriously endangers human health. Although radical surgical resection is an important measure in the treatment of gastric cancer, quite a few patients are still likely to have tumor recurrence after D2 radical resection, which makes the prognosis of advanced gastric cancer patients unsatisfactory (25). Therefore, over the past 20 years, people have been trying new comprehensive treatment options for gastric cancer. At present, according to the results of ACTS GC and CLASSIC studies, AC after radical D2 surgery can prolong the overall survival of patients with advanced gastric cancer (8, 26–29). However, several studies have shown that NAC can also improve the overall survival rate of patients with advanced gastric cancer compared with surgery alone (30, 31). Based on the MAGIC study (10), perioperative chemotherapy has become the standard in European countries, and based on the recent FLOT4 study, the fluorouracil + leucovorin + oxaliplatin + docetaxel (FLOT) regimen is currently the standard for Western populations (32). In East Asian countries, adjuvant chemotherapy after D2 gastrectomy including S-1 or capecitabine plus oxaliplatin is currently the standard regimen based on the ACTS-GC (8) and CLASSIC (33) trials. In addition, docetaxel + S-1 is also the standard treatment for Japanese patients with stage III gastric cancer based on the JACCRO GC-07 trial (34). Through systematic evaluation and summary analysis of existing RCTs, this study explored the effects of perioperative chemotherapy and postoperative AC on the survival benefits of patients with resectable gastric cancer, and provided a certain theoretical basis for further improving the treatment level of gastric cancer, with a view to prolonging the survival period of patients.

The results of this study showed that NAC significantly prolonged PFS in gastric cancer patients compared to AC, and sensitivity analysis did not detect significant instability. Combined with the results of previous studies, it is suggested that NAC has definite advantages in PFS. However, results of subgroup analysis suggest that female patients are more sensitive to NAC, and male patients may not benefit from NAC. Research results of Xu et al. (35) showed that gender and age may be factors that independently predict the effect of NAC in patients with locally advanced gastric cancer. However, most studies took age and gender as baseline data for comparison, and accurate conclusions could not be drawn. In this study, only 3 RCTs reported gender subgroup data, so the study conclusion may have selective bias, and the effect of gender on the effect of NAC needs to be confirmed by further studies.

In addition, subgroup analysis based on lymph node staging found that cN+ patients were more sensitive to NAC, and cN- patients may not benefit from NAC. Kim et al. (36) followed up 108,731 patients with gastric cancer and found that radical surgery, depth of tumor invasion and lymph node metastasis were three important prognostic factors for gastric cancer. Therefore, if early diagnosis can be made clearly and corresponding regional lymph node dissection can be performed at the same time of radical surgery, the survival rate of patients can be significantly improved, especially the long-term survival rate of patients with stage III gastric cancer can be effectively improved (37). Another prospective study conducted by Siewert et al. (38) also showed that lymph node metastasis is one of the important factors affecting the long-term prognosis of gastric cancer. However, according to an exploratory analysis of PRODIGY, patients with cT4 disease were the ones who benefited most from neoadjuvant chemotherapy, regardless of whether they were lymph node positive or not (39). Therefore, the subgroup analysis of this study found an effect of lymph node on PFS, which may be due to different patient inclusion criteria. RESONANCE (14) included II-III disease regardless of lymph node status, the PRODIGY (22) study included patients with cT2/3 disease only when they were clinically lymph node positive, and RESOLVE (23) included patients with cT4a disease only when they were clinically lymph node positive. This difference suggests that these results need to be further verified. In addition, it should be considered that NAC may cause regression of the tumor itself, which may help relieve symptoms such as abdominal pain and dysphagia that may occur in patients with cT2 or cT3, even if they are cN-.

In addition, this study found that NAC had no significant advantage over AC in OS. This finding is consistent with previous research. Reddavid et al. (40) carried out a systematic review on whether patients with locally advanced gastric cancer could benefit from NAC. The study included 16 RCTs. Results showed that of the 6 well-designed RCTs, only 2 RCTs showed a survival advantage of NAC in the esophagogastric junction tumor subgroup. The efficacy of standardized surgery and appropriately expanded lymph node dissection is even better than that of neoadjuvant therapy. Cai et al. (41) conducted a network meta-analysis that included 33 RCTs (8989 patients) published after 1997. The results showed that perioperative NAC had no survival advantage compared with postoperative chemoradiotherapy, postoperative chemoradiotherapy and preoperative chemoradiotherapy. However, it is worth noting that the sensitivity analysis found significant instability in OS. When the data of Iwasaki (21) were excluded, the difference in OS changed from insignificant to significant, the heterogeneity decreased to 0%, and the conclusion suggested that perioperative chemotherapy had a significant effect in prolonging OS. The reason for this result may be that the two latest long-term follow-up trials, PRODIGY (22) and RESOLVE (23), both found the advantages of perioperative chemotherapy in OS. This result is worth reconsidering the efficacy of perioperative chemotherapy on OS in gastric cancer patients, but it also needs to be confirmed by more RCTs with large sample sizes.

Although this study was unable to conduct subgroup analysis through NAC regimen and cycle number due to insufficient data, it is worth noting that the impact of NAC cycle number and regimen on postoperative survival of gastric cancer patients is still controversial. On the one hand, some people believe that increasing the number of chemotherapy cycles may further shrink the tumor and lower the stage, thereby improving the postoperative survival rate (42). However, on the other hand, some studies have pointed out that too long a chemotherapy cycle may lead to a decrease in the patient’s physical tolerance and an increase in postoperative complications, which may adversely affect postoperative survival (43). SOX regimen has received widespread attention in neoadjuvant chemotherapy for gastric cancer due to its significant efficacy and relatively low toxicity. The results of the RESOLVE study (12) show that for patients with locally advanced gastric cancer, 3 cycles of SOX neoadjuvant chemotherapy before surgery can significantly improve the 3-year DFS and increase the R0 resection rate. Therefore, the SOX regimen is listed as the preferred regimen for distal advanced gastric cancer by China’s gastric cancer-related diagnosis and treatment guidelines and consensus. The XELOX regimen is another commonly used neoadjuvant chemotherapy regimen for gastric cancer. Although it did not show better efficacy than the SOX regimen in some studies, the XELOX regimen is still widely used in clinical practice (44). For patients with gastric cancer whose pathological stage is pII/pIII after D2 radical surgery, the XELOX regimen is recommended as an option for postoperative adjuvant chemotherapy (45, 46). The ECF/ECX regimen (epirubicin + cisplatin + fluorouracil/capecitabine) also has a place in neoadjuvant chemotherapy for gastric cancer (47). However, due to the toxicity of the anthracyclines in the ECF regimen and the limited efficacy of the regimen itself, the ECF regimen is no longer recommended by the Chinese gastric cancer guidelines (48, 49). Despite this, the MAGIC study still confirmed that three courses of ECF before and after surgery can further improve the OS and DFS of patients with locally advanced gastric cancer (50). FLOT regimen (docetaxel, oxaliplatin, and fluorouracil) has been a research hotspot in the field of neoadjuvant chemotherapy for gastric cancer in recent years (51). The results of the FLOT4-AIO study show that compared with the ECF/ECX regimen, the FLOT regimen can further improve the R0 resection rate and pathological response rate, thereby improving the patient’s 5-year OS rate and DFS rate (52). Therefore, the FLOT regimen is also regarded as an effective option for neoadjuvant chemotherapy for gastric cancer (42, 53). In addition, the DOS regimen (docetaxel, oxaliplatin, and Tigeol) showed good efficacy and safety in the Korean PRODIGY study (11), and can be used as one of the recommended regimens for neoadjuvant chemotherapy for locally advanced gastric cancer. Considering that the effect of NAC is affected by many factors, including NAC regimen, number of cycles, tumor type, stage, chemotherapy drugs and doses, postoperative complications of patients, etc., patients should be fully considered when determining the number of cycles of neoadjuvant chemotherapy. Based on the specific situation, a personalized treatment plan can be developed. In addition, studies have shown that the application of the multidisciplinary diagnosis and treatment model (MDT) can also help provide patients with more accurate and effective NAC solutions (54, 55).

However, we must acknowledge several limitations of this meta-analysis. Firstly, none of 7 included RCTs had low risk in the allocation concealment, blinding of participants, personnel, and outcome assessment. Secondly, the RCTs included in our study had different intervention (different NAC strategies and AC strategies), which may be one of the sources of heterogeneity. On the other hand, the definition of PFS varies among different studies, which may also be one of the sources of heterogeneity in this study. Although it was not clearly stated whether positive resection margins was defined as a PD event in the MAGIC, FLOT4, and RESOLVE studies, positive resection margins was listed as a PD event in the FNCLCC and FFCD (56) and PRODIGY (11) studies on PFS, resulting in an early decline in their survival curves. Considering the issue of subsequent treatment after positive resection margins and the fact that the PFS benefit was converted into an OS benefit in the FNCLCC, FFCD, and PRODIGY studies, it seems reasonable to define positive resection margins (including distant metastases diagnosed during or after surgery) as a PD event in the neoadjuvant setting (6, 57). In addition, differences in populations may also be a potential source of heterogeneity in this article, especially differences in tumor stages, which may also affect the therapeutic effect of NAC to some extent.

Thirdly, due to the small number of literature, this study could not obtain enough data to combine surgery-related outcomes and chemotherapy response outcomes. At the same time, due to data limitations, this study did not analyze subgroups of patients with different perioperative chemotherapy regimens, number of cycles, age, and pathological types of tumors. Fourthly, all of the included studies were from Asian countries (including Japan, Korea and China) and the data of European populations were still deficient. Another unavoidable limitation is that the proportion of patients for whom NAC might be recommended who actually receive it is often greater than the proportion of patients who actually start and complete adjuvant chemotherapy in patients for whom NAC is recommended postoperatively because of postoperative complications and decreased performance status. This is not reflected in randomized controlled trials that include patients after surgery because patients who are not suitable for adjuvant therapy due to the above factors would not be included in the trial population. Despite several limitations of this meta-analysis, we conducted the latest meta-analysis of RCTs to compare perioperative versus adjuvant chemotherapy for resectable gastric cancer. Results of this meta-analysis validated the superiority of the NAC for PFS of gastric cancer compared with AC. More large-scale, multi-center, double-blind RCTs are needed to further confirm our findings.

## Conclusion

The results of this study demonstrate that NAC can significantly prolong PFS in patients with resectable gastric cancer compared to AC, and the benefit is more significant in women and cN+ patients. Besides, our analysis indicated that NAC has a potential to improve OS compared with AC. Considering the limitations of this paper, such as small sample size, missing data and regional selectivity bias, more large-scale, multi-center, double-blind RCTs are needed to further compare the efficacy of perioperative versus adjuvant chemotherapy for resectable gastric cancer.

## Data Availability

The original contributions presented in the study are included in the article/supplementary material. Further inquiries can be directed to the corresponding author.
